# Discovering the Phylodynamics of RNA Viruses

**DOI:** 10.1371/journal.pcbi.1000505

**Published:** 2009-10-26

**Authors:** Edward C. Holmes, Bryan T. Grenfell

**Affiliations:** 1 Center for Infectious Disease Dynamics, Department of Biology, The Pennsylvania State University, Mueller Laboratory, University Park, Pennsylvania, United States of America; 2 Fogarty International Center, National Institutes of Health, Bethesda, Maryland, United States of America; 3 Department of Ecology and Evolutionary Biology and Woodrow Wilson School, Princeton University, Princeton, New Jersey, United States of America; Massachusetts Institute of Technology, United States of America

## Phylodynamics: The Discovery Phase

The advent of extremely high throughput DNA sequencing ensures that genomic data from microbial organisms can be acquired in unprecedented quantities and with remarkable rapidity. Although this genomic revolution will affect all microbes alike, our focus here is on RNA viruses, as the rapidity of their evolution, which is observable over the time scale of human observation, allows phylodynamic inferences to be made with great precision. In the foreseeable future it is likely that complete genome sequencing will become the standard method of viral characterization, providing the highest possible resolution for phylogenetic studies. The rapidity with which genome sequence data were generated from the ongoing epidemic of swine-origin H1N1 influenza A virus [1] is testament to the power of this technology.

Understandably, pathogen discovery is a major focus of this new-scale genome sequencing [2]. It is now possible to sequence the entire assemblage of viruses in a particular tissue type or host species [3]–[5], as well as all those viruses that are associated with specific disease syndromes [6],[7]. In essence, this new era of metagenomics constitutes a crucial taxonomic discovery phase in virology and epidemiology that allows the genetic characterization of new viruses within hours of their isolation.

Assembling an inventory of viruses that may emerge in human populations is of major importance to public health and to students of biodiversity. However, it is only the first step in developing a full quantitative understanding of the processes that shape the epidemiology and evolution—the phylodynamics—of RNA virus infections [8]. To achieve this goal, we argue here that the field of viral phylodynamics requires its own discovery phase; that is, a comprehensive and quantitative analysis of the interaction between the ecological and evolutionary dynamics of all circulating RNA viruses from the molecular to the global scale. Such a marriage of phylogenetic and epidemiological dynamics is currently only potentially possible for the select few human viruses for which large genome sequence datasets have been acquired, such as HIV and influenza A virus, and even here fundamental gaps in our knowledge remain (see below). Indeed, it is striking that so few complete genome sequences are currently available for viruses whose epidemiological dynamics are known in exquisite detail, such as measles [9],[10]; these sequences have been so sparsely sampled in both time and space that a full phylodynamic perspective has not yet been achieved. We contend that a better understanding of RNA virus phylodynamics will allow more directed attempts at pathogen surveillance, facilitate more accurate predictions of the epidemiological impact of newly emerged viruses, and assist in the control of those viruses that exhibit complex patterns of antigenic variation such as dengue and influenza. Just as PCR and first-generation DNA sequencing ushered in the science of molecular epidemiology, so next-generation sequencing may herald the age of phylodynamics. Box 1 lists a number of key questions that can be addressed within this phylodynamics research program.

**Box 1. Key Research Questions in RNA Virus Phylodynamics**

1. What is the range of phylodynamic patterns observed in RNA viruses? Can they be categorized into specific groups? How do these patterns relate to other “life history” variables exhibited by RNA viruses?
2. What epidemiological and evolutionary processes give rise to these phylodynamic patterns? What generalities can be drawn?
3. How commonly does natural selection (compared to neutral evolutionary processes) determine the population dynamics of pathogens? On what scale does natural selection act? How does viral immune escape reduce herd immunity at the population level and allow the persistence of viral lineages in epidemic troughs?
4. What is the range of spatial patterns exhibited by RNA viruses? What epidemiological factors are responsible for these patterns?
5. How do different viral species (various respiratory viruses, for example) interact in host immunity?

A number of important advances are needed to meet our goal of a comprehensive catalog of the diversity of phylodynamic patterns in RNA viruses. Because answers to many of the most interesting research questions depend on sufficiently large sample sizes, we require large numbers of sequences that have been rigorously sampled according to strict temporal, spatial, and clinical criteria, and that as much of these data are publicly accessible as possible. A phylodynamic analysis has little value unless viral genomes are sampled on the same scale as the epidemiological processes under investigation.

The only acute virus for which a suitably expansive genome dataset currently exists is influenza. In this case, the >4,000 complete genomes generated under the Influenza Genome Sequencing Project [11] have provided important new insights into the evolution and epidemiology of this major human pathogen [12]. To highlight one key insight here, these genome sequence data have revealed that multiple lineages of influenza virus are imported and circulate within specific geographic localities (even within relatively isolated populations), generating both frequent mixed infections [13] and reassortment events [14]. Even so, the sampling of these genome sequences (and associated epidemiological covariates) may not be dense enough to fully capture spatial dynamics [15]. There is also a marked absence of samples from asymptomatically infected patients (or those with mild disease), so it is impossible to link genetic variation to clinical syndrome. Such a bias against viruses sampled from individuals with asymptomatic infections is a common problem in molecular epidemiology.

## Epidemiological Factors

**Figure 1:**
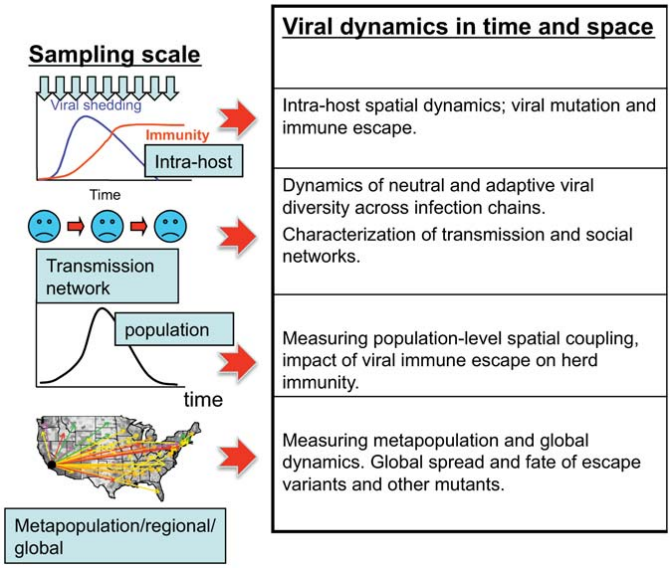
Sampling scales for acute RNA viruses and the associated phylodynamic processes that viral genome sequence data and host sampling can elucidate.

It is also clear that for many RNA viruses we need to better understand a number of key epidemiological factors, such as the interaction between local persistence, epidemic dynamics in both time and space, the impact of measures to control the spread of infection, and the consequences of adaptive evolution in those viral genes that interact most intimately with the host immune response. It is instructive to imagine the ideal database for addressing these issues. In the case of acute infections, the goal would be to collect four parallel datasets on the appropriate scale of interest during outbreaks (Figure 1). This database would comprise, first, *epidemic dynamics in time and space*, ideally at a comparable or higher frequency than the generation time of individual infections. Second, and in parallel, our ideal study would collect *viral genome sequence data* at these time points, sampling both within and among infected hosts. Both disease incidence data (bolstered by contact tracing) and viral sequence data furnish information on the transmission network traced by an outbreak. Third, we would need to know the underlying *contact network* of susceptible individuals, which serves as fuel for the epidemic. This is a difficult structure to measure directly, although novel measurements of human interactions are increasingly shedding light on the problem [16]. Finally, measurements of the *immunity structure* of our contact network [17]—reflecting the past history of the virus in the population—are key for understanding both the dynamics of epidemic spread and the evolutionary pressures that shape virus diversity.

The outbreak of foot-and-mouth disease (FMD, an RNA virus infection of cattle) in the UK in 2001 resulted in a database that is arguably closest to our ideal on the epidemiological scale [18],[19]. Notwithstanding a variety of gaps in data from the epidemic [20], it is one of the most well-documented large outbreaks in terms of the availability of spatiotemporal incidence data in parallel with contact tracing and the underlying spatial pattern of the susceptible farms as a measure of the contact network. In addition, analyses of viral sequences from relatively small samples of farms have drawn important conclusions about epidemic spread and allowed the testing of new methods to recover the spatiotemporal patterns written into sequence data [18],[20]. Importantly, samples exist from over half the ∼2,000 confirmed infected premises in 2001: sequencing whole FMD virus genomes from these samples would provide a vast resource for basic and applied developments in integrating epidemiological and phylogenetic information to dissect spatiotemporal spread. We suggest that achieving this task would be a huge contribution to understanding the phylodynamics of acute viruses. Another virtue of animal infections like FMD is that the relationship between the determinants of viral variability within and between hosts can also be dissected by experimental infections (see [21] for another example).

A parallel limitation of many phylogenetic approaches to viral epidemiology is that they have often proceeded in the absence of the necessary metadata, such as the precise time and place of sampling or those that relate to clinical syndrome [22]. A perhaps more challenging goal for phylodynamics is therefore to integrate phylogenetic patterns with other biological variables, such as the nature of antigenic variation, the capacity for drug resistance, or the clinical syndrome of the host, as well as the spatial host network data outlined above. Cohort studies may be the most productive way to link genomics with epidemiological variables.

**Figure 2:**
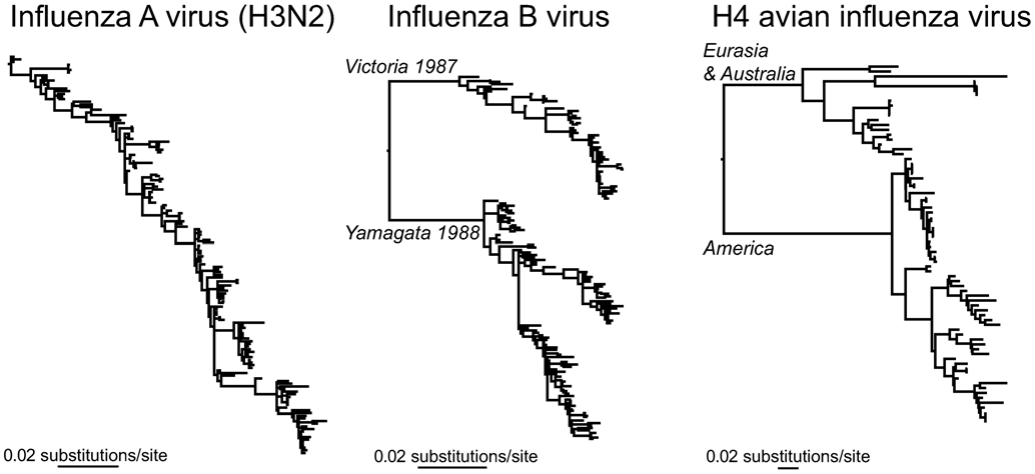
Phylodynamic patterns of human and avian influenza viruses. The left diagram shows the phylogeny of the hemagglutinin (HA) gene of human H3N2 influenza A viruses sampled between 1985 and 2005, revealing the “ladder-like” branching structure indicative of antigenic drift. By comparison, the phylogeny of the HA gene of human influenza B virus sampled over the same interval (center diagram) shows the co-circulation of the antigenically distinct “Victoria 1987” and “Yamagata 1988” lineages, as well a shorter length from root to tip, reflecting a lower rate of evolutionary change. Finally, the phylogeny for the HA gene of H4 avian influenza virus (right diagram) reveals the deep geographic division between the Eurasian and Australian versus North American lineages of this virus.

The lack of a synthesis of phylogenetic and phenotypic/epidemiological data is reflected in the current debate over the mode of antigenic evolution in human influenza A virus. Although it has long been known that the hemagglutinin (HA) and neuraminidase (NA) proteins of human influenza A virus evolve by strong natural selection to evade the host immune response—a process commonly called antigenic drift [23],[24]—the precise mechanisms by which such drift occurs are uncertain. From a phylodynamics perspective, the key observation is that over long time periods a single lineage of HA sequences from subtype A/H3N2 influenza viruses links epidemic to epidemic [23], although intensive sampling has revealed that single populations may harbor far higher levels of genetic diversity [25]. Rather different phylodynamic patterns are seen in other influenza viruses, including those sampled from birds (Figure 2). Three models have been proposed to explain the distinctive phylodynamic pattern observed in human A/H3N2 viruses: (i) that there is short-lived cross-immunity among viral strains [26], (ii) that the HA evolves in a punctuated manner among antigenic types that are linked by a network of neutrally evolving sites [27], and (iii) that the virus continually reuses a limited number of antigenic combinations [28].

To determine which combination of these models best explains influenza phylodynamics will require more expansive genome sequence data, as well as focused sampling and epidemiological surveillance in Southeast Asia, which is likely the global source population for the virus [29]. More importantly, it is also crucial that these phylogenetic data are combined with detailed, spatiotemporally disaggregated antigenic information. Indeed, it is remarkable that despite the abundance of information on the antigenic characteristics of individual influenza viruses, most notably through the use of the hemagglutinin inhibition (HI) assay [17], these data have not been routinely linked to phylogenetic information. It is clear that both antigenic and phylogenetic analyses would greatly benefit from each other.

## New-Generation Computational Tools

Another important challenge for phylodynamics is to match the remarkable ongoing developments in genome sequencing technology to the increase in the power of the computational tools available to analyze these sequence data. Crucially, in phylogenetics, the size of the space of possible trees increases faster than exponentially with the number of sequences, such that the availability of datasets comprising thousands of complete genomes [30] presents a major combinatorial problem. This problem creates a growing discrepancy between our ability to generate genome sequence data and our capacity to analyze them using the most sophisticated methods. Redressing this balance should be the major goal of bioinformatics in the future; and in fact some progress has been made recently [31].

**Figure 3:**
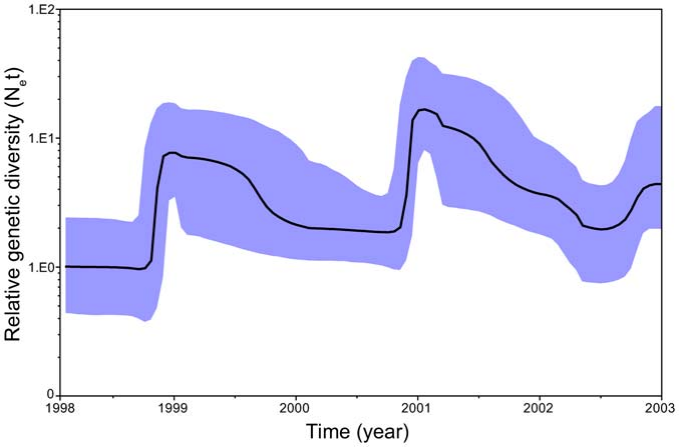
Fluctuating genetic diversity of influenza A virus. The figure shows a Bayesian skyline plot of changing levels of genetic diversity through time for the HA gene (165 sequences) of A/H3N2 virus sampled from the state of New York, US, during the period 2001–2003. The *y*-axes depict relative genetic diversity (*N*_e_ *t*, where *N*_e_ is the effective population size, and *t* the generation time from infected host to infected host), which can be considered a measure of effective population size under strictly neutral evolution. Peaks of genetic diversity, reflecting the seasonal occurrence of influenza, are clearly visible. See [30] for a more detailed analysis.

It is also clear that improvements need to be made to the methods that are available to analyze genome sequence data. A powerful set of research tools in this area comprises those based on coalescent theory, as this provides a natural link between the analysis of epidemiological and phylogenetic patterns [8],[32]. In particular, the coalescent allows the demographic characteristics of viral populations (particularly population size and growth rate) to be inferred directly from gene sequence data. Coalescent analyses are especially powerful in the case of RNA viruses, because their rapid evolution means that temporal and spatial dynamics are discernable over the period of human observation [33] and can in theory be combined with time series epidemiological data. However, currently available coalescent methods are restricted by the limited scope of demographic models and their inability to fully incorporate spatial information. In particular, most acute RNA viruses have complex population dynamics that combine distinct periods of growth and decline. The most commonly used phylodynamic tool available in such cases is the Bayesian skyline plot (and the related Bayesian “skyride” [34]), which represents a piecewise graphical depiction of changes in genetic diversity through time [32]. In the case of neutral evolution, such changes in genetic diversity also reflect underlying changes in the number of infected hosts. Although the Bayesian skyline plot can reveal unique features of epidemic dynamics (Figure 3) [30], precise estimates of parameters such as population growth rate are not yet possible.

The coalescent methods commonly used to study RNA virus evolution focus largely on temporal dynamics (a natural function of the rapidity of viral evolution), with little consideration of patterns of spatial diffusion. Although these phylogeographic patterns are becoming increasingly well described for RNA viruses [35], few methods effectively recover the spatial component in genome sequence data. For example, commonly used parsimony-based approaches consider a single phylogenetic tree without an explicit spatial model (see, for example, [36]). In addition, these methods usually describe the place of origin and direction of spread of viral lineages without formal tests of competing spatial hypotheses. As a specific case in point, although gravity models (in which patterns of viral transmission reflect the size of and distance between population centers) have been applied successfully to morbidity and mortality data from human influenza A virus to describe its spread across the United States [37], they have yet to be interpreted within a phylogenetic setting. A clear push for the future should therefore be the development of coalescent tools that integrate the analysis of spatial and temporal dynamics within a single framework, with a focus on those that combine phylogenetic data and information on the dynamics of the host contact network of susceptible, infected, and immune individuals.

## Looking beyond the Consensus Sequence

The vast majority of studies of RNA virus evolution undertaken to date, particularly of those viruses that cause acute infections, rely on the analysis of consensus sequences in which the nucleotide shown for any given site is the most common among all the genomes within a patient. Although the use of consensus sequences is adequate for many aspects of molecular epidemiology, in which complete genomes may suffice to determine even tight transmission chains [20], there is growing evidence that key evolutionary processes occur beyond the consensus. In particular, extensive intra-host gene sequencing has revealed the existence of minor viral subpopulations within individual hosts that are not detected by consensus sequencing and that are sometimes of great phenotypic importance [38],[39]. Given the intrinsically high mutation rates of RNA viruses, as well as the immense size of intra-host populations, such extensive genetic and phenotypic diversity is only to be expected.

A full description of the extent and structure of intra-host viral genetic variation is critical for understanding evolutionary dynamics, informing on such issues as the frequency of mixed infection, and hence the degree and extent of cross-immunity; the frequency with which antigenic variants are produced and whether antigenic evolution can occur on the time scale of individual infections; and the size of the population bottleneck that might accompany inter-host transmission. As a case in point, it is commonly assumed that viruses experience a severe population bottleneck as they are transmitted to new hosts, a phenomenon that greatly restricts the power of natural selection to fix advantageous mutations. Although this assumption appears to be true in some cases [40], whether this is a general property of RNA viruses is unclear; the evidence that multiple viral lineages can be transmitted among hosts argues against a narrow bottleneck in all cases [41]. To more accurately determine the size of the transmission bottleneck, analyses of intra-host genetic diversity along known transmission chains will be essential. On a larger scale, it is unclear whether phylodynamic patterns differ within and among hosts, and whether any differences among these scales of analysis are qualitative or quantitative.

Intra-host sequence data are also essential for understanding the process of cross-species virus transmission and emergence. Key parameters in determining whether a virus will adapt successfully to a new host species include the extent of intra-host genetic diversity, the fitness distribution of the mutations produced, and how many of these mutations will assist adaptation to new host species [41]–[43]. No such data are available for any acute RNA virus, so testing models for viral emergence is difficult. We believe, however, that understanding the mechanics of this adaptive process is at least as important as surveying for new emerging viruses.

## Challenges for the Future

Our discussion has highlighted a number of key challenges for a successful phylodynamic research agenda. These challenges comprise data, theory, and methodological issues, and are briefly summarized as follows. First, with respect to data, it is clear that more genome sequences must be acquired and with increased temporal and spatial precision. For example, wherever possible, GenBank records should contain the exact day and precise latitude and longitude of sampling. In addition, it is essential that these sequence data be linked with the relevant metadata, such as the associated clinical syndrome and (if applicable) measure of antigenicity. Similarly, it is essential that equivalent genome sequence data be acquired from multiple time points within individual hosts. Second, in terms of theory, it is crucial that we fully integrate patterns of viral evolution across multiple epidemiological scales, from within hosts, to local outbreaks, and on to global pandemics. Although the coalescent is hugely useful in this respect, it is essential that its theoretical framework be extended to incorporate models of population growth and decline that most accurately reflect the population dynamics of acute RNA viruses, in particular the dynamics of the susceptible “denominator” that fuels epidemics. Sequencing of all available samples from the UK 2001 FMD epidemic would yield great scientific dividends here. Third and finally, with respect to methodology, new computational tools are needed to rapidly make phylodynamic inferences from genomic datasets that may contain thousands of sequences and that efficiently integrate genomic with other forms of biological data. We hope this review will stimulate research in all these areas.
